# Insufficient LGBTQ+ education across disciplines suggested by national survey of health professionals in training

**DOI:** 10.1371/journal.pone.0316931

**Published:** 2025-01-06

**Authors:** Lexie Wille, Tess Jewell, Atticus Wolfe, Emily Peterson, Aileen Shaughnessy, Cole Roblee, Alex Strader

**Affiliations:** 1 Gender & Sexuality Program, Department of Psychiatry, Columbia University Irving Medical Center, New York, NY, United States of America; 2 University of Wisconsin School of Medicine and Public Health, Madison, WI, United States of America; 3 Department of Public Health, Agnes Scott College, Decatur, GA, United States of America; 4 Dept of Psychological Sciences, Case Western Reserve University, Cleveland, OH, United States of America; 5 Dept of Physician Assistant Studies, Le Moyne College, Syracuse, NY, United States of America; 6 Chicago Medical School, Rosalind Franklin University of Medicine and Science, North Chicago, IL, United States of America; 7 Dept of Physician Assistant Studies, Ohio University, Athens, OH, United States of America; Ascension Sacred Heart Hospital Pensacola, UNITED STATES OF AMERICA

## Abstract

Health professionals often feel underprepared to treat patients who identify as lesbian, gay, bisexual, transgender, and/or queer (LGBTQ+). Additionally, lack of access to professionals who are knowledgeable about LGBTQ+ inclusive care contributes to the myriad of health disparities experienced by LGBTQ+ communities. This cross-sectional survey study explores the preparedness of healthcare profession trainees for caring for LGBTQ+ patients by quantifying the hours and quality of training health profession trainees receive in LGBTQ+ education across disciplines. We surveyed US-based health professionals in training (HPiT) across disciplines (N = 155) on their training programs’ LGBTQ+-specific curricula and educational opportunities. Ordered logistic regression analysis assessed the relationship between the number of hours of LGBTQ+-specific education and other discipline, organization, and individual factors. Respondents reported an average of 4.75 (*SD* = 3.04) hours devoted to LGBTQ+-specific education. Physician assistant trainees reported receiving the highest number of hours of LGBTQ+-specific education (*M* = 6.63, *SD* = 1.98), followed by psychology (*M* = 5.30, *SD* = 3.54), medical (*M* = 5.12, *SD* = 3.38), nursing (*M* = 4.17, *SD* = 3.28), and trainees in other health fields (*M* = 3.88, *SD* = 2.47). Across all disciplines, trainees rated their LGBTQ+-specific education on average as “good”. Despite rising awareness, the quantity and quality of dedicated LGBTQ+-specific education remains concerningly low across all measured disciplines and US regions. Future research must investigate strategies to overcome common barriers to increasing LGBTQ+ education in health professions training by maximizing the impact of limited hours through integrating LGBTQ+ content into existing materials, supporting trainee leadership, and implementing institutional support for educators teaching LGBTQ+ content. Regulatory bodies must reconsider the current guidance for LGBTQ+ education quantity and quality to advise institutions on best-practice guidelines to prepare trainees for LGBTQ+ patient care.

## Introduction

Despite increasing visibility and conversation surrounding healthcare for individuals who identify as lesbian, gay, bisexual, transgender, and/or queer (LGBTQ+), health disparities and reports of negative healthcare experiences persist [1]. Negative healthcare experiences include reports of misgendering, heterosexist assumptions, and a lack of knowledge about LGBTQ+ health concerns on the part of health professionals [2–8]. For LGBTQ+ patients, negative healthcare experiences contribute to worse health outcomes over time [6, 7, 9]. Corroborating patient reports, health professionals report feeling underprepared to care for LGBTQ+ patients [10–12]. This lack of preparation contributes to patient mistreatment and poorer health across the LGBTQ+ population.

Previous research suggests that the education provided to health professionals in training (HPiT) is not sufficient to help trainees feel comfortable treating LGBTQ+ patients [13], thus contributing to LGBTQ+ patients’ negative healthcare experiences. In 2011, medical school leadership (deans and administrators) reported a median of 5 hours of LGBTQ-related content in curricula in the United States (US) and Canada [14]. A 2024 replication of this study indicated an increase in median hours of reported LGBTQ+-specific education time, to 11 hours [15]. Even with this increase of 6 reported hours over the course of medical school, 11 total hours still falls short of the recommendations from experts in the field that medical students should: receive 10 total hours of required LGBTQ+-specific curriculum; engage in 25 hours of supplemental LGBTQ+ education; and work with at least 35 LGBTQ+ patients in a clinical setting throughout their training [16]. When considering the findings from the 2024 survey, it is important to consider how the participant sample could bias the results. Previous surveys of medical students themselves about their LGBTQ+ curricular time contradict the medical school administration’s median estimate of 11 hours. One survey of 940 medical students in the United States in 2020 found that students reported receiving an average of just 2.22 annual hours of LGBTQ+-specific education [16]. While we cannot perfectly compare these different surveys from different time periods, these findings demonstrate the importance of gathering updated perspectives from medical students.

Additionally, research in this area has heavily prioritized medical school, leading to a significant gap in knowledge about the duration and quality of LGBTQ+ health education in other health professional training programs. Limited existing research suggests that similar LGBTQ+ training gaps exist in other health professional disciplines such as nursing, psychology, physician’s assistants, physical therapy, and dental training programs [17–24], but few studies have compared across disciplines [13, 21, 23]. Studies that have compared across disciplines have found significant differences across disciplines in terms of reported attitudes, knowledge, and preparedness, with trainees in general reporting low clinical preparedness to work with LGBTQ+ patients and poor perceptions of their formal training in LGBTQ+ healthcare [13, 23].

Our team of HPiT sought to survey other US-based HPiT about their educational experiences related to caring for LGBTQ+ patients, to add to the literature in this understudied area. An updated analysis of current educational practices is needed in order to develop effective curricula and recommendations for health professional training programs. The present study reports the quality as well as the quantity of LGBTQ+ education across health professional disciplines. By improving LGBTQ+-specific education and clinical experiences in health professional training programs, the next generation of health professionals could prevent the neglect, dehumanization, and frustration that has thus far predominated healthcare experiences for LGBTQ+ patients [2–5, 7, 9].

## Methods

### Study design

This study was determined to be exempt from review by the institutional review board at The University of Texas at Austin (STUDY00003195). The data were analyzed anonymously, and thus, participant consent was not required to be obtained. Our research team is composed of HPiT who are members of the HPiT Curriculum Reform Committee of GLMA: Health Professionals Advancing LGBTQ+ Equality (GLMA, formerly known as the Gay and Lesbian Medical Association). Team members developed and distributed a mixed-methods online survey that investigated LGBTQ+ health education among HPiT programs across healthcare disciplines in the US. Data was collected from November 2022 to November 2023. Participants were asked to complete an online survey via Qualtrics that assessed perceptions of their training program’s LGBTQ+-specific curricula and educational opportunities. Participation was optional, anonymous, and not incentivized. Survey materials did not include any mention of GLMA: Health Professionals Advancing LGBTQ+ Equality, and rather listed only the lead authors’ institution and PI information. Participants reviewed all study information on the first page of the Qualtrics survey, and were alerted that by clicking the “Proceed” button, they were offering their general consent to participate in the study.

### Subjects and setting

Participants were recruited using snowball [25, 26] and respondent-driven sampling. Members of the research team sent recruitment emails that clearly identified the survey as being organized by the GLMA HPiT Curriculum Reform Committee and stated that the survey was related to LGBTQ+-specific health information in either the program they attended as trainees or the program where they worked as educators. Recruitment emails were shared with the research teams’ professional networks (e.g., colleagues at HPiT programs), professional email listservs (e.g., GLMA member email list), and contacts of other organizations (e.g., LGBTQ+ affinity groups at individual programs). Eligibility criteria included: (a) current trainees, at any point in training, in healthcare fields such as nursing (including advanced nursing degrees), pharmacy, medicine (including allopathic and osteopathic medical doctorate and physician assistant), dentistry, psychology/mental health counseling (including masters-level counseling students, and clinical, counseling, and school psychology Ph.D. students) rehabilitation (including physical, occupational, and speech therapy), vocational programs (such as pharmacy technician), or other clinical training programs (b) 18-years and older, (c) consent to survey participation, and (d) speak English fluently.

### Survey instrument

The survey was developed based on input from an interdisciplinary team of HPiT, including psychology, physician assistant, and medical trainees. After consenting to the Qualtrics survey, participants answered questions to confirm their age and degree paths to determine inclusion or exclusion in the study as an adult HPiT. If they fit the inclusion criteria, participants were brought to the appropriate survey. The HPiT survey consisted of 33 questions, though not every question was provided to every respondent due to branching logic.

Survey items focused on quantity, subject matter, and learning experiences related to LGBTQ+ health included in participants’ curriculum. For example, respondents were asked an open-ended question, “How many hours of your curriculum so far have been devoted to LGBTQ+-specific topics, if you had to estimate?” Respondents were also asked for details about their training, such as the type of program they attend, the state where the program is located, and their current year in the program. After reshaping the data for analysis, the final analytic sample included N = 155. Descriptive and statistical analysis techniques were completed using Stata version 17 [27]. See S1 Appendix to review the full survey.

### Variables

#### Dependent variable

The reported number of hours in each respondent’s curriculum devoted to LGBTQ+-specific topics was coded as a single numeric value. If a respondent had entered a range, their response was coded as the average time in the range (for example, 5–10 hours would be coded as 7.5 hours). Blank responses were counted as missing and removed from the analysis. Because hours were not normally distributed, the hours variable was winsorized to the 90^th^ percentile, affecting 4 outlying values [28].

#### Independent variables

Respondents indicated their gender as (a) cisgender man, (b) cisgender woman, (c) transgender man, (d) transgender woman, (e) nonbinary, gender non-conforming, and agender, and (f) another gender. A binary variable was created from the gender variable to measure transgender status and included those who identified as transgender, nonbinary, gender non-conforming, agender, question, and/or another gender. Respondents indicated their sexuality as (a) straight, (b) gay or lesbian, (c) bisexual, (d) pansexual, (e) asexual, and (f) queer or another sexuality. A binary variable for LGBTQ+ identity was created from the gender and sexuality variables that included transgender, lesbian, gay, bisexual, pansexual, asexual, and queer respondents. Respondent discipline included those in MD/DO, nursing, psychology (master’s and doctoral levels), physician assistant, and other programs.

#### Covariates

Respondents indicated the state within the US wherein their program resides. States were coded according to their region (Northeast, Southeast, Midwest, West, and Southwest). Respondents indicated their current year in their program (e.g., first-year, second-year, third-year, fourth-year, fifth year and greater). The survey also measured racial and ethnic identity. For the purpose of the analysis, racial categories were created for racial and ethnic identities that are under-represented in medicine (URiM) (e.g., American Indian or Alaska Native; Black or African American; Hispanic, Latino, or of Spanish Origin; or Native Hawaiian or Other Pacific Islander identities), and those who are over-represented in medicine (e.g., White and Asian identities) in accordance with the Association of American Medical Colleges (AAMC) [29].

### Analytic strategy

Baseline descriptive statistics and linear regression models were compiled using Stata version 17 [27]. The relationship between the reported hours of LGBTQ+-specific education and trainee discipline was assessed using linear regression models (Model 1). In model 2, the relationship between reported hours of LGBTQ+-specific education, trainee discipline, and trainee LGBTQ+ identity was assessed while controlling for the respondent’s year in their current program. Model 3 assessed the relationships in model 2 while also controlling for region and URiM identity. Statistical significance was considered at alpha = 0.05.

## Results

The median time to complete the survey was 8.70 minutes. Survey respondents were excluded from analyses if they did not respond to 60% or more of the quantitative survey measures (N = 75, 30%). Frequency of missing responses on each variable was less than 10%.

### Respondent characteristics

Eligible responses were obtained from 155 HPiT. Respondents included medical (MD/DO) students (N = 53, 34%), physician assistant (PA) students (N = 32, 21%), psychology and mental health students (N = 28, 18%), nursing students (N = 21, 14%), and students in other disciplines (N = 21, 14%). Other disciplines included students in pharmacy, dentistry, podiatric medicine, rehabilitation (including physical, occupational, and speech therapy), genetic counseling, athletic training, clinical research, and biomedical sciences. Seventy-six (50%) HPiT attend programs in the Midwest, 33 (22%) in the West, 21 (14%) in the Northeast, 20 (13%) in the Southeast, and 2 (1%) in the Southwest. Eighty-five (55%) individuals self-identify as LGBTQ+. Thirty-three (21%) self-identified as an URiM race. Demographics of the respondent sample are displayed in Table 1.

**Table 1:** Demographics by discipline.

	Full Sample (N = 155)		MD/DO (N = 53)		Nursing (N = 21)		Psychology and mental health (N = 28)		Physician Assistant (N = 32)		Other (N = 21)	
Variables	*M* or %	(SD)	*M* or %	(SD)	*M* or %	(SD)	*M* or %	(SD)	*M* or %	(SD)	*M* or %	(SD)
Gender												
Cisgender man	17%		25%		5%		7%		22%		19%	
Cisgender woman	64%		58%		76%		50%		72%		71%	
Transgender^‡^	19%		17%		19%		43%		6%		10%	
Sexuality												
Straight	42%		25%		57%		36%		66%		43%	
Gay or lesbian	10%		11%		10%		0%		9%		19%	
Bisexual	19%		26%		14%		14%		22%		10%	
Pansexual	2%		0%		5%		7%		0%		0%	
Asexual	7%		11%		0%		7%		0%		14%	
Queer or another sexuality	20%		26%		14%		36%		3%		14%	
Hours	4.75	3.04	5.12	3.38	4.17	3.28	5.30	3.54	6.63	1.98	3.88	2.47
Year in program			1.47	1.54	1.37	1.12	1.52	1.28	0.97	0.59	1.29	0.90
Region												
Midwest	50%		58%		25%		30%		88%		24%	
Northeast	14%		8%		0%		11%		6%		57%	
Southeast	13%		15%		10%		26%		3%		10%	
West	22%		17%		65%		30%		3%		10%	
Southwest	1%		2%		0%		4%		0%		0%	
Race												
White and Asian American	79%		74%		90%		75%		81%		81%	
URiM	21%		26%		10%		25%		19%		19%	
Quality	2.35	1.19	2.21	1.07	2.42	1.21	2.55	1.23	2.63	1.30	2.33	1.43

^‡^Includes those who identified as transgender, nonbinary, gender non-conforming, agender, questioning and/or another gender

### Primary aims

#### Time spent on LGBTQ+ health education

Across all disciplines, HPiT reported receiving a mean of 4.75 hours (*SD =* 3.04) of LGBTQ+-specific education. Physician assistant students reported receiving the highest number of hours of LGBTQ+-specific education (*M* = 6.63, *SD* = 1.98), followed by psychology students (*M* = 5.30, *SD* = 3.54), medical students (*M* = 5.12, *SD* = 3.38), nursing students (*M* = 4.17, *SD* = 3.28), and students in other health fields (*M* = 3.88, *SD* = 2.47).

#### Predictors of time spent on LGBTQ+ health education

Table 2 displays the findings from the linear regression models assessing characteristics that predict time spent on LGBTQ+ health education. Reported hours of LGBTQ+-specific education received thus far was associated with the respondent’s year in their training program and LGBTQ+ identity. Prior to controlling for covariates, LGBTQ+ identity had a strong negative association (β = -1.41, p<0.01) and year in training program had a moderate positive association (β = 0.51, p<0.05) (model 2). When controlling for discipline, region, and race in model 3 (r^2^ = 0.14), LGBTQ+ identity (β = -1.43, p<0.01) and year in training program (β = 0.58, p<0.05) remained significant predictors of reported hours of LGBTQ+ education (model 3, Table 2).

**Table 2:** Linear regression models.

Outcome: reported hours of education or training that included LGBTQ+ people or issues	Model 1		Model 2		Model 3	
	F = 1.06		F = 3.01		F = 1.98	
	R-squared = 0.03		R-squared = 0.11		R-squared = 0.14	
Variables	Coeff.	(SE)	Coeff.	(SE)	Coeff.	(SE)
Discipline						
MD/DO [Ref.]	-	-	-	-	-	-
Nursing	-0.96	0.78	-1.32	0.82	-1.38	0.88
Psychology and mental health	0.18	0.71	-0.09	0.70	0.02	0.73
Physician Assistant	-0.50	0.68	-0.75	0.70	-0.69	0.74
Other	-1.24	0.78	-1.44	0.77	-0.75	0.88
LGBTQ+ identity			-1.41**	0.51	-1.43**	0.52
Year in program			0.51*	0.23	0.58*	0.24
Region						
Midwest [Ref.]					-	-
Northeast					-1.35	0.89
Southeast					-0.71	0.83
West					0.08	0.73
Southwest					0.07	2.17
Race						
White or Asian American [Ref.]					-	-
Racial identity that is under-represented in medicine (URiM)					0.72	0.64

N = 155 Model 1

N = 152 Model 2

N = 150 Model 3

*, **, *** denote significance at the P≤0.05, P≤0.01, and P≤0.001, respectively.

### Secondary aims

#### Quality of LGBTQ+ education

Across all HPiT, when asked how they would rate the quality of the LGBTQ+-specific education in their health professional program, respondents gave an average rating of 2.35 (*SD* = 1.19), which corresponds to “good” (on a scale of 0 = “poor” and 4 = “excellent”). When looking across disciplines, physical assistant trainees had the highest rating of their curriculum (M = 2.63, SD = 1.30), followed by psychology (*M* = 2.55, *SD* = 1.23), nursing (*M* = 2.42, *SD* = 1.21), trainees in other health fields (*M* = 2.33, *SD* = 1.43), and medical students (*M* = 2.21, *SD* = 1.07).

#### Sources of education

**Fig 1:**
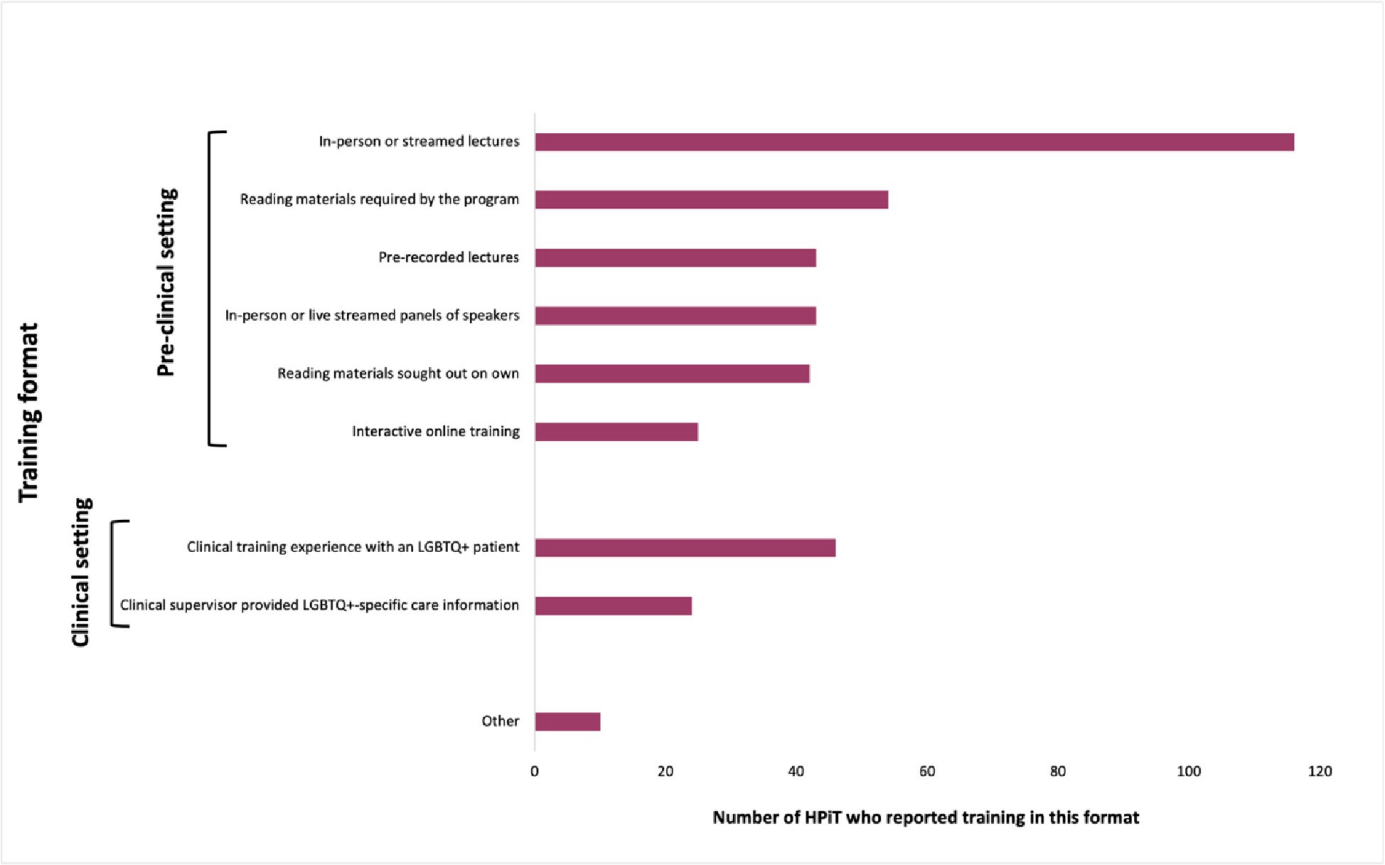
In-person or streamed lectures were the most common training format in which HPiT learned about LGBTQ+ health.

The majority (*N* = 116, 75%) of HPiT reported learning about LGBTQ+ health through in-person or streamed lectures. Forty-six (30%) HPiT reported clinical training experience with an LGBTQ+ patient and 24 (15%) reported that a clinical supervisor provided LGBTQ+-specific care information. Forty-two (27%) HPiT reported learning about LGBTQ+ health from reading materials they sought on their own. The training formats and frequencies at which HPiT reported learning from them are displayed in Fig 1.

## Discussion

The results of this exploratory study indicate an overall lack of progress in LGBTQ+ healthcare education across disciplines, despite expert recommendations calling for at least 10 hours of required curriculum for proficiency for medical students [16]. Respondents’ reported hours of LGBTQ+ education do not differ significantly by discipline after controlling for LGBTQ+ identity, year in program, region, and race, demonstrating that insufficient inclusion of LGBTQ+ health topics is prevalent throughout many HPiT programs and disciplines. Results suggest that the quantity and quality of LGBTQ+-specific HPiT education remains inadequate, and new strategies are needed to ensure that patients have access to high quality healthcare.

Additionally, respondents that identified as LGBTQ+ were more likely to report fewer hours of LGBTQ+-specific education in their training program compared to their heterosexual, cisgender counterparts. LGBTQ+ trainees may be better equipped to judge the quantity and quality of LGBTQ+-specific education compared to their heterosexual, cisgender counterparts. Literature across disciplines indicates a similar pattern wherein those who belong to the LGBTQ+ community are more sensitive to the accuracy and inclusivity of education materials, making them more cognizant of gaps [13].

Importantly, it should be noted that trainee-estimated hours is not a perfect proxy for true time spent on LGBTQ+ education content, and hours may ultimately not be directly correlated with trainees’ eventual sense of preparedness or comfort in working with LGBTQ+ patients. Estimated curricular hours also do not fully account for active learning experiences or clinical encounters with LGBTQ+ patients or clients, and do not represent the quality of the curricular content itself. Thus, we aim to use the results of this survey of multidisciplinary HPiT to call for an increase in focused, high-quality LGBTQ+-specific educational content and for an increase in mandatory clinical encounters with LGBTQ+ patients to improve trainees’ sense of preparedness, rather than increasing curricular time for increased times’ sake. Among the HPiT survey respondents in the present study, just 46 (30%) reported receiving clinical training experience with at least one LGBTQ+ patient. This result may reflect the amount of time dedicated to clinical activities that trainees have experienced thus far in their training program, or may suggest that HPiT may not receive sufficient opportunities to engage with LGBTQ+ care in the clinical setting. Direct care experiences with LGBTQ+ patients provides trainees with the opportunity to learn directly from these communities in order to best meet their needs.

Our study has several strengths. First, this study was developed, fielded, and analyzed by a team of HPiT representing several programs and disciplines, ensuring that diverse trainee experiences and perspectives were taken into account. Second, we obtained a diverse sample, especially geographically, as we obtained responses from HPiT from all geographic regions of the continental United States. Third, we had high completion rates–the frequency of missing responses on each variable was less than 10%. Fourth, whereas previous reports on LGBTQ+ health professions education originated with surveys of program leaders (e.g., medical school deans), our findings indicate the number of hours recalled by trainees, which may be subject to less social desirability bias than administrators. Lastly, our study’s cross-disciplinary and cross-program approach was a strength, as previous work in this area has typically focused on one institution at a time, or sampled a few institutions at a time within a specific discipline. Our team’s approach helped to reduce potential programmatic bias, as the state of LGBTQ+-specific education at one institution is not necessarily representative of the state of LGBTQ+-specific education for that discipline nationally.

In addition to increased high-quality curricular time, it is also important that LGBTQ+-specific education be integrated longitudinally throughout training and include training in relevant clinical skills [30]. Several studies demonstrate the benefits of active learning (compared to the traditional lecture format) in trainee education. Active learning could include integration through case study examples, standardized patients, clinical contact hours, clinical observation, and including these identity demographics in assessment tools. A study of postgraduate resident physicians at one hospital in Boston Massachusetts found a small but significant association between curricular hours and comfort providing care to LGBTQ+ patients, but a significant positive association between patient exposure and comfort providing care to LGBTQ+ patients, suggesting that LGBTQ+ patient exposure may actually be more effective at increasing providers’ comfort than curricular hours alone [31].

HPiT may also benefit from increased opportunities to learn directly from LGBTQ+ community members. A model program intervention conducted at Harvard Medical School was able to successfully incorporate LGBTQ+ community members’ lived experiences and opinions into undergraduate medical education through a series of community panel events, standardized patients, and Community Advisory Groups [32]. Another study of a Community Forum on Transgender Healthcare conducted in Louisville Kentucky found similar positive results by bringing together transgender community members with local healthcare professionals who discussed and made recommendations to improve transgender healthcare in the area [33]. These LGBTQ+ community member-informed interventions provide a guideline for other professional training programs who may wish to use similar structures to incorporate community-informed curricular improvements.

Additionally, HPiT programs should structure opportunities for trainees to act as leaders in health advocacy for LGBTQ+ care. Trainees who value LGBTQ+-competent care may be unsatisfied with the lacking education received through their program and use their personal time to work with community organizations, research labs, or clinics to expand their knowledge of LGBTQ+ patient care. Without adequate education, many HPiT rely on education from sources outside their program to learn about LGBTQ+ health; this pattern is particularly prevalent in LGBTQ+ HPiT groups. While self-driven learning is valuable, the quality and accuracy of this information is no longer under the purview of regulatory bodies and may encourage inaccurate or outdated practices. Additionally, relying on LGBTQ+ care to be learned through self-driven tactics contributes to the division and unequal education across the trainee population, meaning some trainees will be drastically more prepared to care for LGBTQ+ patients compared to others. Education programs are tasked with preparing all trainees with the ability to care for LGBTQ+ patients, not just those with the internal motivation to seek individual opportunities. By encouraging trainees to bring their expertise into the classroom and clinical learning spaces, programs increase the learning opportunities for all trainees while guiding the knowledge gained in external opportunities for accuracy and utility. Furthermore, formal educators (e.g., faculty and deans) should structure opportunities for trainees to take on active leadership roles to advocate for LGBTQ+ representation to advance their scholarship. For example, trainees with advanced knowledge of LGBTQ+ patient care may be invited to serve on curriculum review committees, partner with teaching faculty on presentations, or incorporate their knowledge into learning spaces more broadly.

Finally, educational regulatory agencies should enact guidelines for LGBTQ+ education for HPiT across disciplines. While the AAMC published guidelines recommending the incorporation of LGBTQ+-specific content into undergraduate medical education in 2014 [34], other disciplines have not yet established suggested minimums for LGBTQ+-specific education while in training [35]. Training programs may fail to adapt curricula and clinical structures unless changes and improvements are made mandatory. Previous research suggests that didactic hours are an important part of LGBTQ+-specific health professional training, but are not sufficient alone, and clinical exposure and practice with LGBTQ+ patients is also crucial [16, 36]. We call on the regulatory bodies of health professions training programs to institute mandatory minimums for didactic hours and patient exposure related to LGBTQ+ health for HPiT, in order to improve the care experiences and overall health of LGBTQ+ patients.

## Limitations

Our study must also be considered in the context of its limitations. Notably, LGBTQ+-specific instructional hours are difficult to quantify, and are likely subject to report bias. Previous research in this area notes that respondents’ attitudes towards LGBTQ+ people and the mental association between training and comfort/preparedness can both influence individuals’ estimate of instructional hours [31]. Future research could better account for holistic training experiences by asking additional questions about individuals’ clinical experiences with LGBTQ+ patients and other non-lecture based learning experiences. Additionally, our study presents a non-probability sample using snowball sampling methodology that may not be generalized to the entire healthcare professional population [26]. Our survey was also disseminated through LGBTQ+-affiliated health professional networks and colleagues, and thus both LGBTQ+-identified trainees and trainees interested in LGBTQ+ health were likely overrepresented. This overrepresentation could potentially lead to selection bias, where individuals who are LGBTQ+ themselves or more supportive of LGBTQ+ individuals were more likely to complete the survey. Lastly, because the LGBTQ+ and allied communities are an invisible minority group, we are unable to provide representation data across disciplines or regions. Despite these limitations, this study presents clear patterns across disciplines and identities that warrant action to improve educational standards to prepare future professionals.

## Conclusions

Despite the expanding literature on the gaps in care and health disparities faced by LGBTQ+ communities, the amount of time dedicated to LGBTQ+-specific education for HPiT may remain insufficient. Every health professional discipline requires improvement to prepare HPiT to care for LGBTQ+ patients. More comprehensive studies are needed to determine the degree of sufficiency of the curriculum as well as how LGBTQ+ patient exposure may impact learning. Future studies also need to address the efficacy of curriculum interventions to enhance LGBTQ+ health knowledge across all HPiT programs, as well as to determine the impact of these interventions on downstream LGBTQ+ patient care experiences and health outcomes. Finally, transitioning this research into practical strategies is vital for encouraging appropriate care for LGBTQ+ patients by preparing trainees to become knowledgeable and inclusive professionals.

## Supporting information

S1 File (XLSX)

S1 Appendix Survey questions. (DOCX)
