# Supplementary material for: Insufficient LGBTQ+ education across disciplines suggested by national survey of health professionals in training
Source: PLoS One. 2025 Jan 6;20(1):e0316931. doi: 10.1371/journal.pone.0316931 (PMC11703085; doi:10.1371/journal.pone.0316931)
Supplement: S1 Appendix — (DOCX) [file pone.0316931.s002.docx]

Appendix

**Survey Questions**

**What health professional degree are you pursuing at this time?**

Advanced nursing degree - NP or CRNA

Clinical doctorate such as Pharm.D.

Doctor of Osteopathic Medicine - DO

Doctor of Medicine - MD

Doctor of Dental Surgery – DDS

Doctor of Medicine in Dentistry – DMD

Masters in a mental health/counseling related field - LCSW, LPC, LCPC, LMFT, LPCC, LMHC

Nursing - BSN, RN, MSN

Ph.D. in Psychology

Psy.D. in Psychology

Physician assistant/associate - PA-C

Ph.D. or equivalent in basic or biomedical sciences (molecular biology, chemistry., etc.)

Rehabilitation professionals - DPT, OT, SLP

Vocational program - (e.g., pharmacy tech, CNA, EMT, medical tech)

Health professional degree not listed here, please list: ___

**Do you plan to specialize in a particular discipline after completing your health professional degree? If so, please describe your planned specialty.**

[open text box]

**In what U.S. state or territory is your health professional program located?**

[drop down state menu]

**What year/stage are you currently in your health professional training program? (e.g. 2^nd^ year Ph.D. student, 1^st^ year resident, etc.)**

[open response box]

**Do you identify as a member of the LGBTQ+ community?**

No

Yes

**We would now like to ask you a few questions about your demographic information. These questions will help us to better understand our data. Please note that to ensure your privacy and confidentiality, we will only present this information in aggregate in any future publications of the data. All demographic questions are optional and you may decline to answer.**

**How do you currently identify in terms of your gender identity? (please check all that apply)**

Agender

Cisgender man

Cisgender woman

Genderfluid

Genderqueer

Nonbinary

Questioning

Transgender man

Transgender woman

Two-spirit

Another identity not listed here (please specify):

**How do you currently identify in terms of your sexual orientation?**

Asexual

Bisexual

Gay or lesbian

Pansexual

Queer

Straight/heterosexual

Another identity not listed here (please specify):

**Which categories describe your race/ethnicity? Please choose all that apply.
A space is available after each category if you would like to add more detail to describe your race and ethnicity.**

American Indian, Alaska Native, Indigenous, or First Nation

Asian or Asian American

Black or African American

Hispanic, Latino/a/x. Chicano/a/x, or Spanish Origin

Middle Eastern or North African

Native Hawaiian or Pacific Islander

White

A race, ethnicity or origin not listed here (please specify):

**In your pre-health professional training thus far, have LGBTQ+-specific topics or information been covered in the curriculum?**

No, not at all

Yes, for less than one hour

Yes, for between 1 and 5 hours

Yes, for more than 5 hours

**Which of the following LGBTQ+-specific topics have been covered in your curricula thus far?** (Please check all that apply)

Overview of different sexual orientations, including terminology

Overview of different genders/gender identities/gender modalities, including terminology

Sensitive history taking for LGBTQ+ patients/clients

Sexual health for LGBTQ+ patients/clients

Social determinants of health relating to LGBT care

Gender-affirming care for transgender patients (surgical, medical, and/or social)

LGBTQ+ care for the elderly

LGBTQ+ care for adolescents

Overview of field-relevant comorbidities common in LGBTQ+ populations

**Please describe additional topics and curricula or expand on above selected curricula:**

[open text box]

[IF YES to “have LGBTQ+-specific questions been covered in the curriculum”] **How have LGBTQ+-specific training experiences been delivered to you in your training program? Please check all that apply.**

In-person or live streamed lectures

In-person or live streamed panels of speakers

Pre-recorded lectures

Interactive online training modules

Reading materials that were required by my program

Reading materials that I sought out on my own

A clinical training experience where I saw LGBTQ+ patients

A clinical supervisor provided me with LGBTQ+-specific care information

Other, please specify:

[IF YES to “have LGBTQ+-specific questions been covered in the curriculum”] **How would you rate the quality of LGBTQ+-specific education you have received so far in your health professional program?**

Poor

Fair

Good

Great
Excellent

[IF YES to “have LGBTQ+-specific questions been covered in the curriculum”] **How would you describe the strengths of the LGBTQ+-specific education you have received thus far in your health professional program?**

[open response box]

[IF YES to “have LGBTQ+-specific questions been covered in the curriculum”] **Given the LGBTQ+-specific education you have received thus far in your health professional training program, in what ways could your program’s LGBTQ+-specific education be improved?**

[open response box]

[IF YES] **Given the LGBTQ+-specific education you have received thus far in your health professional training program, what are the strengths of the LGBTQ+-specific education provided by your health professional program?**

[open response box]

**How important do you think LGBTQ+-specific health information is to your health professional practice or specialty?**

Not at all important

-

Somewhat important

-

Very important

**Please select the choice below that estimates the amount of LGBTQ+-specific education you have received in your health professional training program relative to the amount that you think you should receive.**

1 - I have received very little LGBTQ+-specific education information relative to the amount that I would like to receive.

2

3

4 – I have received some LGBTQ+-specific education information, but not as much as I would like to receive.

5

6

7 - I have received all of the LGBTQ+-specific education information that I would like to receive.

[IF YES to “have LGBTQ+-specific questions been covered in the curriculum”] **How many hours of your curriculum so far have been devoted to LGBTQ+-specific topics, if you had to estimate?**

[blank text box]

[IF YES to “have LGBTQ+-specific questions been covered in the curriculum”] **When LGBTQ+-specific topics or information have been covered in your curriculum, have they been** **condensed into discrete lectures or day(s)where LGBTQ+ topics are the only focus, or** **integrated longitudinally throughout a variety of lectures and curricula? Please expand upon your response.**

[blank text box]

[IF YES to “have LGBTQ+-specific questions been covered in the curriculum”] **Would you like to say more about the structure of LGBTQ+-specific integration in your health professional curriculum thus far?**

[open response box]

[IF YES to “have LGBTQ+-specific questions been covered in the curriculum”] **When LGBTQ+-specific topics or information have been covered in your curriculum, are those lectures or modules mandatory for students in your degree program?**

No, none have been mandatory

Some have been mandatory, some have been optional or recommended

All have been mandatory

[IF NO to “have LGBTQ+-specific questions been covered in the curriculum”] **Why do you think LGBTQ+-specific information has NOT been included in your health professional training curriculum thus far? More specifically, what barriers do you think have gotten in the way of receiving LGBTQ+-specific information in your education program?**

[open response box]

**[ALL RESPONDENTS] What, if any, efforts have you engaged in to promote LGBTQ+ healthcare education in your health profession training program?**

[open text box]

[ALL RESPONDENTS] **How prepared do you feel to treat cisgender LGB people after you complete your health professional degree due to the education you received in your health** professional training?

Not at all prepared

Somewhat prepared

Moderately prepared

Very prepared

[ALL RESPONDENTS] **How prepared do you feel to treat transgender and gender diverse people after you complete your health professional degree due to the education you received in your health professional training?**

Not at all prepared

Somewhat prepared

Moderately prepared

Very prepared

[IF NO, NOT PREPARED] **What makes you feel unprepared to treat cisgender LGB people after you complete your health professional degree? What would you need to feel more prepared?**

[open response box]

[IF NO, NOT PREPARED] **What makes you feel unprepared to treat transgender and gender diverse people after you complete your health professional degree? What would you need to feel more prepared?**

[open response box]

[IF YES, PREPARED] **What experience in your training program helped you the most to feel prepared to treat cisgender LGB people?**

[open response box]

[IF YES, PREPARED] **What experience in your training program helped you the most to feel prepared to treat transgender and gender diverse people?**

[open response box]

[IF YES, PREPARED] **What would you want to learn more about to continue to feel prepared to treat LGBTQ+ people in your clinical practice?**

[open response box]
